# New Frontiers in Management of Early and Advanced Rectal Cancer

**DOI:** 10.3390/cancers14040938

**Published:** 2022-02-14

**Authors:** Jordan R. Wlodarczyk, Sang W. Lee

**Affiliations:** Division of Colorectal Surgery, Norris Cancer Center, Keck School of Medicine, University of Southern California, 1441 Eastlake Avenue, Suite NTT-7418, Los Angeles, CA 90033, USA; jordan.wlodarczyk@med.usc.edu

**Keywords:** rectal cancer, TAMIS, watch and wait, HIPEC, transanal TME, neoadjuvant, robotic TME, metastasectomy

## Abstract

**Simple Summary:**

Rectal cancer has the capacity to present in a variety of forms. Depending on subtle differences in the characteristics of the tumor, it is possible for the treatment protocol to vary drastically. There are many tools available to optimize patients’ outcomes when treating rectal cancer of these various stages. Advances in early-stage, local presentation of rectal cancer focus on minimally invasive endoluminal surgery. Lesions completely responding to neoadjuvant chemoradiation non-operative surveillance have been explored. For rectal cancers patients with problematic pelvic anatomy, new platforms for resection, such as transanal total mesorectal excision and robotic total mesorectal excision, have been introduced. Late solid organ and peritoneal metastasis, originally thought to be a terminal disease, have undergone recent advances in hepatic and pulmonary metastasectomy, cytoreductive surgery, and intraperitoneal chemotherapy. Understanding these various therapeutic interventions will pave the way for improved patient outcomes.

**Abstract:**

It is important to understand advances in treatment options for rectal cancer. We attempt to highlight advances in rectal cancer treatment in the form of a systematic review. Early-stage rectal cancer focuses on minimally invasive endoluminal surgery, with importance placed on patient selection as the driving factor for improved outcomes. To achieve a complete pathologic response, various neoadjuvant chemoradiation regimens have been employed. Short-course radiation therapy, total neoadjuvant chemotherapy, and others provide unique advantages with select patient populations best suited for each. With a clinical complete response, a “watch and wait” non-operative surveillance has been introduced with preliminary equivalency to radical resection. Various modalities for total mesorectal excision, such as robotic or transanal, have advantages and can be utilized in select patient populations. Tumors demonstrating solid organ or peritoneal spread, traditionally defined as unresectable lesions conveying a terminal diagnosis, have recently undergone advances in hepatic and pulmonary metastasectomy. Hepatic and pulmonary metastasectomy has demonstrated clear advantages in 5-year survival over standard chemotherapy. With the peritoneal spread of colorectal cancer, HIPEC with cytoreductive therapy has emerged as the preferred treatment. Understanding the various therapeutic interventions will pave the way for improved patient outcomes.

Rectal cancer (cancer within 15 cm from the anal verge) affects approximately 737,000 new patients per year worldwide [1]. With its wide range and early age of presentation, it is important to understand the alterations in treatment with each stage of presentation. Tumor stage and location drive the management principles of rectal cancer. With surrounding peri-rectal structures vital to the gastrointestinal and genitourinary systems, minor local invasion can have devastating effects on normal physiologic function. Important advances in understanding tumor biology and effective chemotherapy and radiation therapy have led to critical improvements in organ and sphincter preservation rates. Improvements in these therapies have even led to protocols for the avoidance of surgery altogether. It is the purpose of this review article to present a clear and concise discussion on the different treatment options for rectal cancer of various stages and locations.

## 1. Early Rectal Cancer (Pre-Malignant Polyps, Carcinoma In-Situ, and T1 Carcinoma)

Early-stage rectal cancer includes a variety of malignant and pre-malignant lesions, but what is critical to understand is that the disease process has spread no farther than the muscularis propria. Because of the need for preoperative staging of rectal cancer to guide therapy, stage 1 disease, by definition, possesses no evidence of extraluminal spread on radiologic evaluation. Different modalities are utilized for the assessment of the depth of tumor invasion, but with findings of shallow depth of invasion and no clinically apparent lymphatic spread, the treatment of early-stage rectal cancer can be directed towards endoluminal curative resection. The location of the lesion in relation to the sigmoid takeoff and the valves of Houston can pose difficulties with obtaining the visualization and angulation needed for the surgical resection of the polyp, carcinoma in situ, or cancerous lesion. What is crucial when considering which technique to employ is the consideration of what technique will remove the cancerous lesion in its entirety.

### 1.1. Transanal Endoscopic Microsurgery (TEM) and Transanal Minimally Invasive Surgery (TAMIS)

Low rectal lesions not suspicious for metastatic spread have traditionally been treated with sphincter sparing transanal endoluminal excision. This transanal endoscopic excision first utilized a conventional anal retractor for the visualization of the tumor or suspicious polyp. This minimally invasive endoscopic technique allows for the miniatous of the rectal reservoir and pelvic innervation and can work to avoid symptoms of low anterior resection syndrome, which have been reported to be as high as 25–80%.

This transanal endoscopic excision was limited by the small endoluminal working space and lack of reach of traditional tools. Buess et al., in 1985, first introduced TEM as a way to obtain surgical resection of these high rectal lesions and reported success in lesions as high as 18 cm from the anal verge in his preliminary group of 33 patients [2]. This was quickly followed by his follow-up experience with 75 patients, which reported the accurate and non-invasive resection of sessile adenomas and early carcinomas up to 25 cm from the anal verge [3]. As the surgical envelope has been pushed, TEM and TAMIS have become viable options for the treatment of lesions in the upper rectum. Published data on TEM highlight its ability to provide a more complete specimen than traditional transanal excision with improvements in the fragmented specimen (0% vs. 37%), negative resection margins (98% vs. 78%), and lower recurrence rate (8% vs. 24%) [4]. These findings were similar to TAMIS when compared with traditional transanal excision as well with a 4% fragmentation rate, 6% microscopic margin positivity, and a 2% recurrence rate. While there is a lack of robust randomized control trials for TAMIS- and TEM-facilitated removal of proximal lesions of the upper rectum, TAMIS and TEM’s reach of three times the traditional conventional anal retractor emphasize their role as an important tool in the colorectal surgeon’s armament.

The indication for the utilization of TAMIS and TEM relies on a discussion with a multidisciplinary team along with a detailed discussion with the patient regarding the risks and benefits of offering local excision without lymphadenectomy. Accurate radiologic or endoscopic assessment of the tumor depth and local and distant metastatic spread is vital for making a sound oncological decision. They are most appropriate for the resection of pre-cancerous lesions, carcinoma-in situ, and superficial (SM1) cT1N0 lesions with favorable clinical and histologic features. Lesions most successfully treated by TAMIS include all lesions (<3 cm) that are typically limited to <30% of the bowel wall circumference. While 3 cm is the recommended size criteria for lesions amenable for TAMIS removal, a small case series demonstrating the success of surgical resection >3 cm has been recorded [5,6,7,8,9,10]. TEM and TAMIS involve local excision of the tumor with a full-thickness excision containing the mucosa, submucosa, and muscularis propria. Care is taken to stay in the mesorectal envelope and not injure the peritoneum. If injury to the peritoneum is encountered, the peritoneum is repaired in a single layer incision. Over one-centimeter gross margins on the oncologic sample are recommended for resection with a deep margin of two millimeters [11].

TAMIS, while able to remove lesions through a minimally invasive approach, has the weakness of not adequately staging the mesorectal lymph nodes. Various classification systems have been identified to stratify lesions based on their depth of invasion of the submucosa, such as the Haggitt and Kudo classifications, for malignant pedunculated and sessile polyps. The ability of these classification systems to delineate high-risk polyps (Haggitt stage 3/4 and SM 2/3) from low-risk polyps (Haggitt stage 1/2 and SM 1) can provide estimates of the risk of concurrent lymphatic spread with malignant polyps, thus guiding the appropriate surgical therapy. Risks of lymphatic spread in SM2/3 sessile polyps and Haggitt 3/4 pedunculated polyps range from 5.8% to13.0%, which represents an unacceptable risk in some patients [12]. TEM and TAMIS have been selectively studied in rectal cancer with a depth of invasion of T2. Lezoche et al., in 2008, compared their local failure rates, rates of distant metastasis, rates of local and distant failure, and survival in patients with T2 rectal cancer treated with either a total mesorectal excision or by TEM. With a median follow-up time of 84 months, all of these parameters of oncologic success were similar between the two groups demonstrating TEM’s early success in eliminating select local disease confined to the rectum [13]. These findings were later contradicted by a large meta-analysis of a retrospective study on TEM and TAMIS vs. radical resection. Data on recurrence rates were conflicting with 50% (six studies), demonstrating higher local recurrence rates among patients who underwent local excision. Two additional studies showed no increase in local recurrence rates among patients who underwent local excision of T1 lesions but a significantly higher local recurrence rate among those who underwent local excision of T2 lesions. In 7 of 15 studies, long-term survival was reduced compared with that of patients who underwent radical resection. This prompted the recommendation that TAMIS may be indicated for selected patients with T1 lesions [14]. Important risk factors for metastatic spread are poorly differentiated tumors, tumor budding on tissue biopsy, lymphovascular invasion, and suspicious lymph node morphology on staging MRI or endoscopic ultrasound. The American Society of Colon and Rectal Surgeons recommends each case be carefully considered for their candidacy based on their histologic findings [15]. In the case of suspected cT1 without LN metastases, initial transanal full-thickness excision to assess the depth of invasion is reasonable. Patients who are good candidates for abdominal surgery found to have SM2 or SM3 lesions should undergo resection with LN dissection. Similarly, patients found to have T2 cancers should undergo resection with LN dissection.

### 1.2. Robotic Transanal Minimally Invasive Surgery

With the rise in surgical technology surrounding the da Vinci Surgical System (Intuitive Surgical, Inc., Sunnyvale, CA, USA) and the increased degrees of freedom of the instruments, various studies have been undertaken to identify these platforms’ feasibility facilitating a more effective repair. All have demonstrated no technical difficulties applying the da Vinci Surgical System to the TAMIS setting, but no randomized clinical trials have directly compared robotic TAMIS to traditional TAMIS with laparoscopic tools [16,17]. Additionally, endoscopic submucosal dissection (ESD), an advanced colonoscopic procedure selectively performed by specialized gastroenterologists and colorectal surgeons, can potentially treat lesions with very superficial submucosal invasion (SM1 < 1000 micrometer invasion) in the middle and upper rectum. The technology behind this is relatively novel when compared to TEM and TAMIS, and its application specific to rectal lesions has no clinical practice recommendations [15,18]. Long-term survival rates after ESD for SM1 colon cancer have been demonstrated to be equivalent to that of the anatomical surgical resection. Similar to TAMIS, success with ESD requires careful patient selection [19,20].

## 2. Locally Advanced Rectal Cancer (Stage 2–3 Disease)

### 2.1. Adjunctive Chemoradiation Therapy

In cases when rectal neoplasm has advanced beyond the mucosa and submucosa into the peri-rectal tissue or the perirectal or pelvic sidewall lymph nodes, transanal endoscopic excision is unable to control the oncologic burden of disease. Because of rectal cancer’s localization to the pelvis, the usage of neoadjuvant chemoradiation has emerged as a powerful tool for disease control. The utilization of a combination chemoradiation therapy in the postoperative setting was first introduced in the GITSG 7175 trial in 1985 and demonstrated significant improvements in 7-year local recurrence vs. surgery without adjunctive therapy, radiotherapy in isolation, and chemotherapy in isolation [21,22,23]. When overall survival was compared, treatment with chemoradiation demonstrated improved overall survival over surgery alone. This highlighted the benefit of combination chemoradiation therapy in the adjunctive treatment of rectal cancer. The shift of chemoradiation therapy to preoperative therapy was the next landmark advance in the multimodal treatment of rectal cancer. The German Rectal Trial in 2004 evaluated preoperative chemoradiation therapy vs. postoperative chemoradiation therapy with a follow-up duration of 45 months and found that 5-year local recurrence, acute grade 3 and 4 chemotherapy toxicity, long-term toxicity, and the incidence of sphincter sparing surgery were all improved in the group who received preoperative chemoradiation therapy [24]. These two trials have contributed to shaping the present-day standard of care for adjunctive therapy in rectal cancers.

### 2.2. Short- vs. Long-Course Radiation Therapy

One recent advance in the adjunctive therapy for rectal cancer centered around the idea that obtaining an expedient potentially curative resection offers the best oncologic outcome over an extended preoperative course of chemoradiation. This minimalization of preoperative adjunctive therapy is postulated to decrease the wait time to oncologic resection, short- and long-term toxicity from chemoradiation, and chemoradiation compliance, without sacrificing long-term survival, local control, and late morbidity. In addition, short-course radiation was touted to be less expensive and a more convenient medium to get patients to their definitive oncologic operation. The Polish I study in 2006 was the first study to compare conventionally fractionated chemoradiation with delayed surgery with short-course irradiation and early surgery [25]. The results demonstrated that acute radiation toxicity was higher in the chemoradiation group without observable effects on 4-year survival, disease-free survival, local recurrence, sphincter preservation surgery, and severe-late toxicity between short-course radiation and long-course chemoradiation. There was a noticeable difference in the rates of the pathologic complete response (pCR), with the conventional long-course radiation group having a pCR of 16.1% as opposed to 0.7% in the short-course group. Circumferential radial margin positivity was also higher in the short-course radiation group (12.9% vs. 4.4%).

Mirroring this study, the TROG 01.04 trial evaluated T3 tumors within 12 cm from the anal verge who received short-course radiation or traditional long-course chemoradiation [26]. Compliance rates were higher in the short-course radiation group than the traditional long-course chemoradiation group, with the long-course chemoradiation group giving rise to more adverse events than its short-course radiation counterpart. These included increases in the rates of radiation dermatitis, proctitis, nausea, fatigue, and grade 3/4 diarrhea in the long-course chemoradiation therapy group. The short-course radiation group had lower rates of permanent stoma and anastomotic breakdowns, while the long-course chemoradiation had higher perineal wound complications. When local recurrence was considered, the rates were similar between the two groups but trended towards lower rates in the long-course chemoradiation group (7.5% vs. 4.4%). This trend was intensified with rectal cancers of the lower rectum, with local recurrence rates for long-course chemoradiation reaching 3% vs. 12% in the short-course radiation group.

The choice to go immediately to surgery after short-course radiation was challenged by the Stockholm III trial with the idea that adverse events could be further minimized by delaying surgery 4–8 weeks after the end of the short-course radiation [27]. Three groups were compared: short-course radiation with immediate surgery within 1 week, short-course radiation with surgery within 4–8 weeks, and traditional long-course chemoradiation. All three groups demonstrated similar rates of local recurrence after 5.2-years of follow-up. When postoperative complications were considered across the three groups, there was no statistical difference in the three groups. When pooled analysis compared patients with short-course radiation with delayed surgery and those with short-course radiotherapy with immediate surgery, short-course radiotherapy with immediate surgery was a risk factor for the development of postoperative complications. This same pooled analysis demonstrated that with short-course radiotherapy with delayed surgery, there was a statistically significant increase in the rate of pCR on the final pathology when compared to short-course radiotherapy with immediate surgery (11.8% vs. 1.7%). This suggests that the delay in surgery may provide additional time for tumor regression more typically attributable to traditional long-course chemoradiation [28,29].

These three randomized, highly powered trials drive the recommendations when attempting to identify patients best suited for short-course radiation. Utilizing these studies and their inclusion criteria, our institutional guidelines maintain that patients with T3 rectal cancer >5 cm from the anal verge, clear circumferential margins on staging MRI/ERUS, or a symptomatic tumor (bleeding or obstructive symptoms) may benefit from short-course radiation and immediate surgery. Those patients with T4, distal tumors (with possible invasion of surrounding structures) will be unlikely to be downstaged to the same degree as short-term radiation with delay or long-course chemoradiation.

### 2.3. Total Neoadjuvant Chemotherapy

During the past decade, the mortality of rectal cancer could be attributed to its high rate of distant metastasis. Despite the initiation of adjuvant chemotherapy in the postoperative setting, patients continue to be more than twice as likely to present with distant recurrence of the disease than local recurrence [30]. While long-course chemoradiation has demonstrated the ability to improve local disease recurrence, there exists a paucity of treatment strategies aimed at controlling obscure micrometastases outside the resection margin. With the idea that persistence of treatment with a longer therapy course could lead to more efficacious tumor regression, total neoadjuvant therapy (TNT) was created for the purpose of promoting the elimination of these deadly local and distant micrometastases. Its advantages include enhanced compliance with planned adjunctive therapy, reduction in tumor stage, and targeting of occult micrometastases with exposure to longer courses of chemotherapy preoperatively.

TNT has been selectively researched and studied until 2018, where Cercek et al. completed a large retrospective cohort comparison of traditional chemoradiation patients and TNT patients (8 cycles mFOLFOX with 5 cycles CAPOX (capecitabine and oxaliplatin) or FLOX (weekly fluorouracil/leucovorin and biweekly oxaliplatin) prior to chemoradiation and surgery and no adjunctive chemotherapy [31]. The TNT group demonstrated a higher rate of minimally invasive surgery (72% vs. 47%) and higher compliance with their chemotherapy regimen (higher average dosages of chemotherapy received, fewer dose reductions, and greater proportions of patients receiving >75% and >90% of their prescribed chemotherapy). TNT patients had higher rates of combined clinical and pathologic complete response (35.7% vs. 21.3%), and the patients managed non-operatively with cCR had no evidence of local tumor regrowth or distance recurrence for at least 12 months. Overall survival and disease-free survival were unable to be assessed by this retrospective study.

The PRODIGE-23 trial was the first large scale trial to prospectively compare traditional long-course chemoradiation with postoperative adjunctive chemotherapy (6 months postoperative modified FOLFOX6 or capecitabine) to TNT (6 cycles neoadjuvant FOLFIRINOX prior to preoperative chemoradiation, followed by surgery and 3 months of adjuvant modified FOLFOX6 or capecitabine) [32]. With the primary outcome of 3-year disease-free survival and secondary outcomes of pCR, overall survival, and 3-year metastasis-free survival, patients were evaluated with 46.5 months follow-up. The TNT group had higher rates of pCR (27.5% vs. 11.7%), higher 3-year disease-free survival (75.7% vs. 68.5%), and 3-year metastasis-free survival (78.8% vs. 71.7%). Overall survival trended towards a significant change (87.7 vs. 90.8%, *p* = 0.077) in the TNT group. This demonstrated TNT’s ability to downstage tumors of the rectum and increase disease-free and metastasis-free survival, supporting the theory that TNT was capable of targeting occult micrometastasis responsible for disease progression after surgery.

With the new advances in short-course radiation therapy and the realization that patients could tolerate shorter, more intense bursts of radiation, TNT incorporating short-course radiation therapy was compared to traditional long-course chemoradiation in 2020 by Hospers et al. and van der Valk et al. [33,34]. In this protocol, patients were randomized to either short-course radiation (5 × 5 Gy over a maximum of 8 days), followed by six cycles of CAPOX of FOLOFX4 consolidative chemotherapy, followed by TME or traditional long-course radiation (28 daily fractions of 1.8 Gy up to 50.4 Gy or 25 fractions of 2.0 Gy up to 50.0 Gy) with concurrent capecitabine followed by TME and adjuvant chemotherapy if required (eight cycles of CAPOX or 12 cycles of FOLFOX4).

Compliance with radiation in the short-course radiation TNT group trended higher than in the traditional chemoradiation group (100% vs. 98%), while compliance with chemotherapy demonstrated the same trend with at least 75% of the protocoled chemotherapy being administered in 84% of the short-course radiation TNT and 58% of the traditional chemoradiation group. Rates of grade 3 toxicity in the preoperative setting were higher in the short-course radiation TNT group (48% vs. 25%) but equalized when postoperative grade 3 toxicities were considered in combination (48% vs. 35%). The frequency and severity of postoperative surgical complications were similar between the two groups as well. At the time of resection, pCR rates were higher in the short-course radiation TNT than the traditional long-course chemoradiation group (27.7% vs. 13.8%). Disease-related treatment failure (23.7% vs. 30.4%) and rate of development of distance metastases (19.8% vs. 26.6%) were lower in the short-course radiation TNT group, while locoregional failure (short-TNT: 8.7% vs. traditional: 6.0%) was similar between the two groups.

Questions center around the order of administration of chemoradiation and chemotherapy in these TNT protocols. Fokas et al. designed and completed a multicenter, randomized, phase II trial evaluating pCR, toxicity, compliance, and surgical morbidity [35]. They compared consolidation chemotherapy after chemoradiation against upfront induction chemotherapy before chemoradiation. They found chemoradiation followed by consolidation chemotherapy had improved rates of pCR (25% vs. 17%), along with better compliance with chemoradiation and less grade 3 or 4 toxicity. Conversely, the compliance with induction/consolidation chemotherapy was higher in the induction chemotherapy group. This was the first study evaluating consolidation vs. induction chemotherapy. While there were significant confounding factors inherent to this study [36], it begins to answer this important question.

Overall, the literature surrounding TNT has built a strong base for use in rectal cancer. With pooled analysis of the existing data demonstrating clear advantages in pCR rates and early disease-free survival with TNT therapy, it represents a promising strategy in locally advanced rectal cancer [37]. Patients with T3-4 tumors with N+ disease (especially N2) at high risk for micrometastasis outside the surgical resection field are strong candidates for TNT therapy. Additionally, patients with low-performance status with low-lying rectal cancers, when offered TNT, have the potential to be locally downstaged and undergo a sphincter sparing surgery or be treated with non-surgical management (watch and wait protocol).

### 2.4. Adjuvant Chemotherapy after Neoadjuvant Chemoradiation

The role of adjuvant chemotherapy in advanced rectal cancer after the administration of neoadjuvant chemoradiation is a topic of controversy. With a third of patients with advanced rectal cancer eventually developing distant metastases, adjuvant chemotherapy is employed to control the spread of disease and eliminate micrometastases still present after surgical therapy [24]. Despite strong recommendations by the National Comprehensive Cancer Network, American Society of Clinical Oncology, European Society of Medical Oncology, and National Institute of Clinical Excellence, the evidence behind and added beneficial effects of postoperative chemotherapy after neoadjuvant therapy and resection is conflicting. Three large, randomized control trials had been completed to address this question. The first two were completed in 2014 by Coinini and the EORTC Radiation Oncology Group. Coinini observed, in a cohort of 634 patients, no difference in five-year overall survival and disease-free survival. In addition, the rates of distant recurrence were similar between the two groups [38]. Concurrently, the EORTC Radiation Oncology Group examined 505 advanced rectal cancer patients receiving preoperative radiotherapy with or without chemotherapy. Between the cohort receiving adjuvant chemotherapy group and the cohort being surveilled, there were no differences in the 10-year disease-free survival and no differences in the incidence of distant metastasis [30]. The third study completed by the Dutch Colorectal Cancer Group a year later mimicked these findings [39], leading to the question “why do we continue to administer adjuvant chemotherapy?”. With adjuvant chemotherapy significantly impacting quality of life [40] and compliance rates with adjuvant chemotherapy poor [41], whether or not to administer postoperative chemotherapy should be given significant thought.

### 2.5. Watch and Wait Protocol

With the advances in neoadjuvant chemotherapy and the high rates of pCR at the time of resection, one question that has been presented in the literature is, do all cases of rectal cancer need oncologic resection? With certain types of chemoradiation-sensitive tumors that show complete clinical response on repeat imaging and endoscopy, is combination chemoradiation therapy adequate to promote lengthy disease-free survival? With postoperative mortality at 6-months ranging from 2% to 8% and as high as 30% in older patients, are we, as surgeons, doing more harm than good with the resection of a disease that has demonstrated a clinical complete response [42]? With the significant intestinal, urinary, and sexual dysfunction that comes with a total mesorectal excision, can this significant postoperative morbidity be avoided?

Ongoing trials on patients presenting with a clinical complete response have been undertaken in order to identify the effect on overall and disease-free survival than non-operative management conveys. Non-operative management, deemed the “watch and wait” protocol, was first introduced by Dr. Habr-Gama out of the University of São Paulo and evaluated patients presenting with a complete clinical response. These patients were treated with non-operative management and follow-up [43]. Patients were defined as complete clinical responders if there was no evidence of disease on clinical, radiologic, and endoscopic studies after the completion of neoadjuvant chemoradiation therapy. Intense surveillance without additional administration of chemotherapy was initiated in this patient cohort to evaluate for both local and distant disease recurrence.

When the patients demonstrating a complete clinical response were compared to non-complete responders receiving surgery and adjuvant chemotherapy, there were no differences in the pre-neoadjuvant staging and disease characteristics. These two groups demonstrated no differences in overall recurrence (watch and wait: 7.0% vs. radial resection: 13.6%) and five-year overall and disease-free survival (watch and wait: 100% vs. radial resection: 88%, 92% vs. radial resection 83%, respectively). On later evaluation at 10-years, the overall and disease-free survival was also similar (watch and wait: 100% vs. radical resection: 97%, watch and wait: 86% vs. radical resection 84%). Only 6.4% of complete clinical response patients treated non-operatively developed systemic recurrence not amenable to curative resection on first presentation. These results were paramount in demonstrating that patients with favorable tumor biology and complete clinical response to neoadjuvant chemoradiation could be non-operatively managed with intensive clinical and radiologic follow-up.

Since 2004, various other studies have come out agreeing with Dr. Habr-Gama’s findings. A recent systematic review and meta-analysis evaluating 23 studies with 867 patients concluded in patients with a complete clinical response managed non-operatively, rates of overall survival and local recurrence are similar to radical resection [44]. While the detentions of complete clinical response and the surveillance protocols differed widely between these studies, the overall clinical benefit of the watch and wait protocol in select patients is strongly apparent. The watch and wait protocol may play an important clinical role in the management of patients with a no residual clinical disease after neoadjuvant chemoradiation. Further clinical questions center around what to do with this clinical complete response. Some studies advocate for the local excision of the primary tumor. To date, no level 1 data exist evaluating whether local excision of the rectal scar in patients with complete clinical response holds clinical benefit over surveillance. Several studies have attempted to evaluate the rates of local recurrence with local excision of stage II and III rectal cancer after TNT. The GRECCAR2 phase 3 trial, which was designed to assess local excision vs. radical resection after good clinical response to neoadjuvant chemotherapy, failed to identify a superiority of local excision over radical resection in terms of side effects and local recurrence but had significant crossover bias in their study design [45]. Meta-analysis has attempted to compare local excision to watch and wait, but with the limitations of the study design, conclusions are difficult to compare [46]. This topic will require future prospective randomized control trials studies to evaluate further before definitive recommendations can be made.

## 3. Surgical Management of Rectal Cancer

Successful oncologic resection in rectal cancer relies on the surgical tenants of wide local excision of the disease to establish clear margin status and resection of adequate lymph nodes for staging [47]. The introduction of, and adherence to, complete total mesorectal excision, which is the resection of the total mesorectal envelope containing the local lymphatic drainage of the rectum [48], has contributed a large part to the improved operative outcomes and long-term survival for rectal cancer patients [49,50]. Notably, total mesorectal excision has produced a notable reduction in rectal cancer recurrence by 30 to 40% [51,52]. The completeness of the mesorectum, as well as other pathologic parameters, such as lymph node harvest and margin involvement, are critical for pathologic staging, prognosis, and subsequent therapy of surgical patients [53,54]. Consequentially, surgical techniques that fail to meet these pathologic standards may be considered inferior to conventional techniques.

The degree of surgical resection depends on the location and circumferential spread of the tumor. The three main resection types for tumors within 15 cm of the anal verge are classified as low anterior resection (LAR) and abdominal perennial resection (APR). Low anterior resection is best utilized for tumors of the rectosigmoid region, where partial or total mesorectal excision is required to obtain an adequate gross margin on the surgical specimen. This surgical distal margin is a continued topic of debate and depends on the height of the tumor in the mesorectal fascia along with its relation to the pelvic floor and anal verge. For proximal, upper rectal cancers tumors in the proximal mesorectal fascial envelope, specific mesorectal excision (partial) is required to obtain a 5 cm gross distal margin. For tumors of the mid-mesorectal envelope, the adequate distal gross margin decreases to 2 cm. Tumors of the distal mesorectal envelope post the greatest difficulty in decision making, as the decision to offer patients sphincter preserving therapy becomes a difficult balancing act with the desire to decrease local recurrence rates. In ultralow anterior resections, the ability to obtain even a 1 cm gross margin in conjunction with a total mesorectal excision has been demonstrated to be an acceptable outcome [55,56,57]. The inability to obtain even a 1 cm gross margin with a total mesorectal excision necessitates the utilization of an abdominal perennial resection (APR) and end colostomy.

Further complicating the decision of what surgery to offer is the functional outcome that ultralow anterior resections can confer on a patient’s quality of life. Low anterior resection syndrome (LARS) consists of a constellation of findings, including fecal urgency, incontinence, increased frequency, constipation, feelings of incomplete evacuation, and bowel-emptying difficulties. Short-term symptoms (within 6–12 months of surgery) are usually due to temporary neorectal irritability in the postoperative period and can resolve with time, while long-term symptoms of LARS (extending more than 12 months after surgery) are more likely permanent. The prevalence of LARS is high, with approximately 80–90% of individuals reporting varying degrees of symptoms [58]. The ability to predict the severity of LARS that a patient will incur with a sphincter preserving operation is still a topic of study in the colorectal landscape and the question of what the overall effect on quality of life, preserving altered, pathologic, and life-altering bowel function as compared to a permanent stoma is vitally important [59].

### 3.1. Robotic Total Mesorectal Excision

Laparoscopic surgical resection for rectal cancers has become increasingly popular over the conventional open technique due to positive patient factors, such as decreased pain, lower morbidity, and faster recovery [60,61]. Additionally, the technique has been found to be non-inferior to open techniques in multiple randomized controlled trials in terms of recurrence, overall survival, and disease-free survival [62,63]. With the increasing prevalence of robotic surgery in the United States and the demonstrated efficacy of laparoscopic surgery in the completion of total mesorectal excision, studies evaluating robotic surgery’s role in the treatment of rectal cancer must be explored.

Utilization of the da Vinci Surgical System (Intuitive Surgical Inc., Sunnyvale, CA, USA) for the completion of a robotically assisted total mesorectal excision has valuable advantages over traditional laparoscopic TME. The use of the robot can allow for an immersive 3-dimensional depth of field, utilization of seven degrees of freedom with its articulating instruments, and a stable camera platform. The first study to evaluate the efficacy of the robot in rectal cancer in a randomized clinical trial setting was completed by the ROLARR Randomized Clinical Trial in 2017 [64]. This was a randomized clinical trial that included 471 patients from 29 different sites across 10 different countries and evaluated all levels of anterior resection (high vs. low) and abdominoperineal resection. These patients were designated to receive robotic-assisted or conventional laparoscopic TME. With the primary outcome of conversion to open total mesorectal excision and secondary outcomes of circumferential resection margin positivity, quality of life, bladder and sexual dysfunction, and oncological outcomes, this study was the first important tool in quantifying the importance of robotic TME. The conversion rate in the robotic cohort was 8.1%, while the conversion rate in the laparoscopic cohort was 12.2%, which demonstrated a distinct trend towards superiority but did not reach statistical significance (adjusted odds ratio = 0.61 [95% CI, 0.31 to 1.21]; *p* = 0.16). In addition, none of the other eight reported prespecified secondary endpoints demonstrated statistical differences between the two cohorts. From this data, they concluded the observed benefit of robotic-assisted laparoscopic surgery for rectal cancer did not appear to justify the additional expense of the robot.

Rouanet et al., in 2018, building on the non-significant trend of the ROLARR study to have less conversion to open TME, evaluated their single-center experience with robotic and laparoscopic sphincter sparing total mesorectal excision [65]. Their data suggested that in sphincter sparing TME, the utilization of the robotic platform resulted in mixed results. There were no differences in overall survival, R1/R0 resection rates, TME grading, length of the distal margin, and circumferential radial margin positivity. There was a difference in the open conversion rate, with robotic TME possessing a conversion rate of 2.0% as opposed to 9.5% found in laparoscopic TME. There were also differences in the median hospital length of stay, with robotic TME patients being more likely to be discharged before postoperative day seven (22% vs. 5.5%). Robotic TME was also associated with a lower number of harvested lymph nodes at pathologic evaluation (15 vs. 19 lymph nodes). From their data, Rouanet et al. concluded robotic surgery, when utilized for patients with difficult anatomy (BMI > 30, low coloanal anastomosis, intertuberous distance under 10 cm, and mesorectal fat area <20.7 cm^2^), contradicted the ROLARR study by demonstrating superior short-term outcomes and overall survival.

Concurrently, in 2018, a smaller randomized control trial out of the National Cancer Center of South Korea highlighted the advantages of robotic TME [66]. Their patient population consisted of cT1-3NxM0 patients who were stratified into robotic TME or conventional laparoscopic TME. Outcomes included quality of the TME sample obtained, circumferential and distal resection margins, the number of harvested lymph nodes, morbidity, bowel function recovery, and quality of life. Their randomized clinical trial demonstrated similar rates of conversion to open surgery (1.5% vs. 0%), distal resection margins (1.5 vs. 0.7 cm, *p* = 0.11), circumferential resection margin positivity (6.1% vs. 5.5%), and rates of complete TME (80.3% vs. 78.1%, *p* = 0.599). Of interest, there were higher harvested lymph nodes (18 vs. 15 lymph nodes, *p* = 0.04), which contradicted Rouanet et al.’s retrospective review. Operative times in the robotic cohort were, on average, 112 min longer, but when evaluated in patients with a BMI over 25, the statistical significance disappeared, suggesting that the robot may play a greater role in patients with higher BMI. With similar results in the two groups, they concluded that equivalent outcomes were achievable when comparing robotic TME and laparoscopic TME.

While the operative outcomes appear similar between robotic and laparoscopic TME, several studies have attempted to pool data to identify overall trends. In one meta-analysis, seventeen retrospective reviews and three randomized control trials were combined for analysis [67]. The collective risk of open conversion statistically favored robotic TME in the retrospective cohort (Odds ratio 0.26 [95% CI, 0.17 to 0.38]), while the randomized control trials trended towards favoring robotic as well (Odds ratio 0.63 [95% CI, 0.35 to 1.13]). Operative time was also statistically longer in both the retrospective reviews and the randomized control trials, with mean differences of 50.35 and 54.4 min, respectively. In the retrospective reviews and the randomized control trials, there were no differences in overall survival, 3-year disease-free survival, local recurrence, lymph nodes harvested, distal margin length, positive circumferential radial margins, and length of stay. With the equivalency of outcomes demonstrated across studies, attention must be turned to the cost implications of performing robotic TME. In a recent meta-analysis combining cost data from robotic and laparoscopic TME, six out of seven studies demonstrated a significantly higher total cost with robotic TME. Four out of four studies in the meta-analysis identified higher operative cost with robotic TME, and zero out of five studies demonstrated no hospital cost savings with robotic TME [68]. Across the board, robotic TME has been associated with increased healthcare expenditure.

With the equivalent outcomes between robotic TME and laparoscopic TME when universally applied to all patients, one must consider the burden to the healthcare system that robotic surgery conveys. Though no significant outcomes have been identified, robotic TME may play a beneficial role in overweight male patients requiring low coloanal anastomosis.

### 3.2. Transanal Total Mesorectal Excision

Traditional transabdominal approaches have been primarily utilized for the surgical resection of low to mid-rectal tumors. With the heterogeneity of patient pelvic anatomy, several MRI-based scoring systems for surgical resection difficulty have been proposed to identify patients in whom a traditional top-down dissection would be difficult [69,70]. Factors identified as causing increased surgical difficulty with the traditional transabdominal approach include high BMI, coloanal anastomosis, short distance between the lowest points of the ischial tuberosities, and a large cross-sectional area of mesorectal fat. In mid- and low-rectal cancer, the forward tapering of the mesorectum in the pelvis and forward angle of the distal rectum facilitate a more difficult surgical dissection leading to a greater propensity for incomplete mesorectal excision and involved circumferential resection margins.

**Figure 1:**
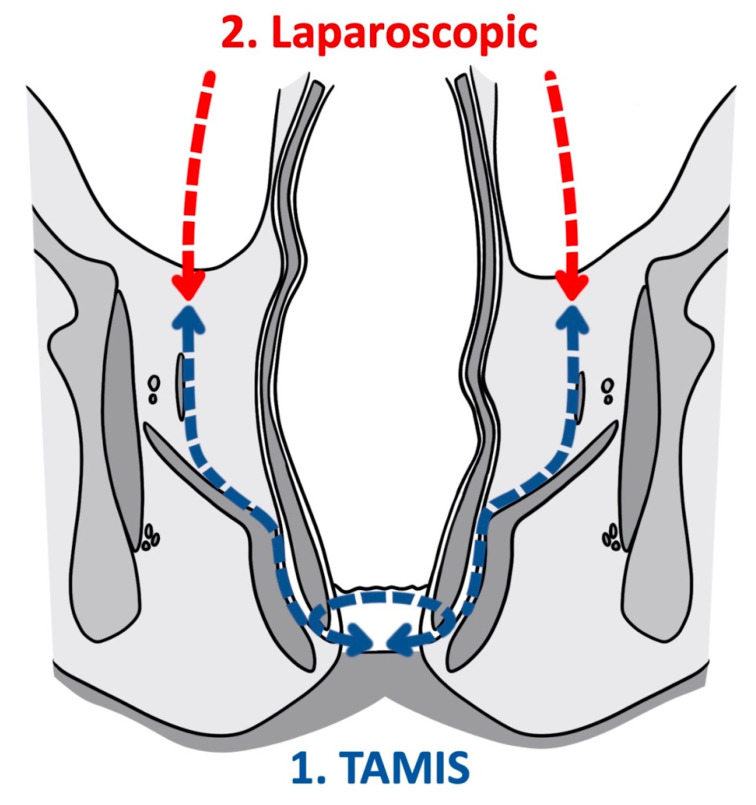
Transanal total mesorectal excision (Coronal).

**Figure 2:**
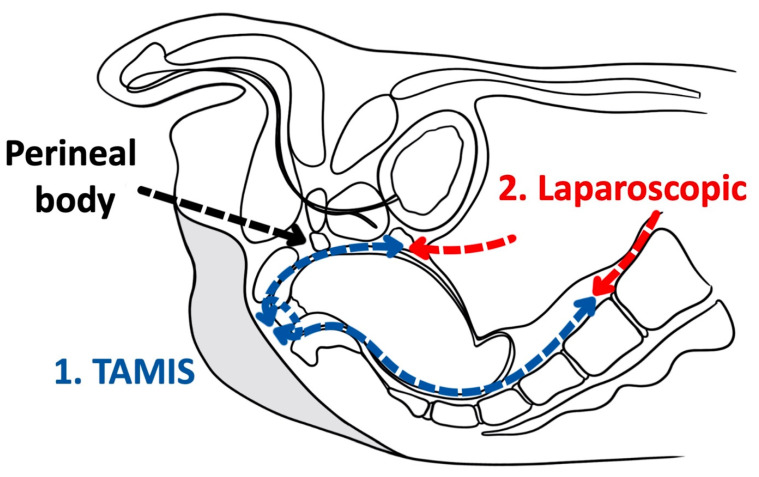
Transanal total mesorectal excision (Saggital).

As a tool for improved visualization with surgical dissection, a combination transanal transabdominal procedure was first described in 1990 [71]. This transanal transabdominal procedure was later adapted by Lacy in 2010 to incorporate a single port laparoscopic platform to facilitate better visualization during transanal TME (TaTME) (Figure 1 and Figure 2) [72]. TaTME has grown in popularity for difficult mid- and low-rectal cancers and can be a powerful tool in sphincter sparing rectal cancer excision. Multiple studies have identified the efficacy of transanal surgical dissection with acceptable postoperative complication rates and TME specimen completeness ranging from 84.0% to 97.1%, but these were small case series and had no direct comparison to the traditional transabdominal approach [72,73,74,75,76]. In the only randomized clinical trial comparing TaTME to traditional transabdominal TME, Denost et al., in 2014, randomized 100 patients with low-rectal cancers (<6 cm from the anal verge) to either TaTME or traditional transabdominal TME [77]. Their primary outcome was a combination of markers for quality oncologic resection (circumferential resection margin, mesorectum grade, and total lymph nodes identified on pathologic evaluation). When compared, the TaTME group and the traditional transabdominal TME group did not differ by any notable descriptive variables. When outcomes were compared, the rate of positive circumferential resection margin decreased significantly when TaTME was employed (4% vs. 18%, *p =* 0.025), with no difference in TME quality, morbidity, number of lymph nodes located at pathologic evaluation. Denost et al. concluded that the TaTME approach reduced the risk of positive circumferential resection margin, as compared with the conventional abdominal dissection in low-rectal cancer, suggesting that perineal rectal dissection could become the new standard in laparoscopic sphincter-saving resection for low-rectal cancer. Denost’s study did not evaluate long-term survival, disease-free survival, or local recurrence of the TaTME procedure.

TaTME is a powerful tool in facilitating increased visualization of the low-rectal dissection of the distal mesorectum that, in cases of unfavorable pelvic anatomy, traditional transabdominal TME cannot provide. To truly identify TaTME as the preferred method for surgical dissection of low-rectal tumors, long-term oncologic outcomes must be compared to traditional transabdominal TME. The COLOR III trial, created by Deijen et al., has been designed to compare local recurrence, disease-free, and overall survival transanal and laparoscopic TME for mid- and low-rectal cancer with the expected end date of May 2025 [78]. This will provide much-needed evidence for the adoption of TaTME as a standard therapy for low-rectal tumors with unfavorable anatomy.

## 4. Distant Solid Organ Metastasis and Peritoneal Carcinomatosis in Rectal Cancer (Stage 4 Disease)

Distant solid organ metastasis and peritoneal carcinomatosis are two presentations of late-stage rectal cancer that carry a poor prognosis. With advances in science and understanding of disease progression, new techniques for the potentially curative resection of these clinical conditions are being explored. With intraperitoneal chemotherapy (IPC) and metastasectomy being introduced for localized disease control, the potential for improvements in overall survival and even the potential for curative resection has been improved [79,80].

### 4.1. Metastasectomy as a Treatment of Isolated Hepatic Solid Organ Metastasis

Despite standardized population screening protocols, approximately 30% of all advanced rectal cancer patients present with distant metastasis and stage four disease [81]. With 5-year survival rates of around 8%, care must be taken to identify patients where additional adjunctive therapy has the potential to extend survival and enhance quality of life [82]. While standard therapy is systemic chemotherapy [83], 20–30% of patients will have potentially resectable lesions at the time of cancer diagnosis. Identifying patients who have the potential to benefit from surgical resection must take into account tumor biology, total oncologic burden, performance status, and other comorbidities. With 5-year survival of metastasectomy for colorectal cancer being ~30% [84,85] vs. untreated potentially resectable liver lesions being <5% [82,86], notable improvements in survival and quality of life can be obtained with surgical resection.

The proper order of surgical resection of these liver metastasis is poorly understood, and the decision is driven by either eliminating all disease or understanding tumor biology and its effect on overall survival. The traditional approach to synchronous rectal cancer liver metastases has been a staged operation where proctectomy is performed followed by hepatectomy. The benefit of this approach is that it allows for the full metastatic load and biological aggressiveness of the tumor to be identified before the morbidity associated with hepatectomy is encountered [87,88,89,90]. Traditionally thought to avoid the increased morbidity and mortality from the combination of the two major operations, several studies demonstrated no increased morbidity or mortality when proctectomy is combined with partial hepatectomy [91,92,93].

The liver-first approach is the next most commonly employed treatment protocol, with its use being most commonly utilized in locally advanced rectal cancer (T3-4Nx disease). In locally advanced rectal cancer, neoadjuvant chemoradiotherapy can potentially take up to 3 months for completion. The localized radiotherapy may allow surgery on the metastatic disease while treatment for the primary lesion is still ongoing. Evidence supporting the liver-first approach is largely based on non-randomized data but remains strongly in support of the viability of the liver-first approach in patients with advanced hepatic metastasis and asymptomatic primary tumors, as the primary determinant of overall survival is the advanced disease burden in the liver [47,94,95,96].

### 4.2. Metastasectomy as a Treatment of Isolated Pulmonary Solid Organ Metastasis

With 1–12% of patients with rectal cancer, developing pulmonary metastasis treatment strategies aimed at improving overall survival must be understood and explored [47]. Since its first utilization by Blalock in 1944, pulmonary resection for colorectal cancer has become a widely accepted treatment for carefully selected patients [97]. The current understanding of patients in which pulmonary metastasis benefits overall survival, presented by the NCCN, include patients who may undergo complete resection based on the anatomic location and extent of disease with the maintenance of adequate function, patients whose primary tumor has been resected for a cure, patients whose resectable extrapulmonary metastases does not preclude resection, and patients whose resectable synchronous metastases can be resected synchronously or using a staged approach [11]. This represents a limited number of patients, with <14% of patients with isolated lung metastasis considered candidates for pulmonary resection [98,99].

These guidelines have limitations due to the design of the previous studies they are based on. Only one study to this point has been designed in the randomized control trial setting due to concerns of a widely accepted treatment practice being withheld from an ideal candidate [100]. This was the PulMiCC trial, and it was ended early due to poor and worsening recruitment. This trial offered contradictory data as survival did not differ between the patients undergoing pulmonary metastasectomy but without full enrollment and powering of the trial, definitive conclusions could not be drawn. The evidence for pulmonary metastasectomy is overwhelmingly supported by other levels of evidence, such as multi-institutional prospective data registries and systematic reviews of non-randomized or non-comparative studies. One registry, the International Registry of Lung Metastases, established in 1991, encompassed a total of 5206 patients who underwent pulmonary metastasectomy. This registry demonstrated a 5-year survival of 36% when pulmonary metastasectomy could achieve complete resection [101]. Numerous studies have mirrored the survival benefit found after pulmonary metastasectomy, with 5-year survival rates ranging from 16% to 64%, with the majority of studies citing 30–40% [102,103,104,105,106,107,108,109,110,111,112,113,114], while possible, concurrent resection of hepatic and pulmonary metastasis carry a poor prognosis with 5-year cumulative survival of 0% [115]. This diverges from pulmonary metastasis observed after hepatic metastasectomy with survival mirroring that is commonly observed in hepatic metastasectomy for colon cancer [110,115]. Several articles have sought to identify differences in the prognosis between pulmonary metastasis from colon and rectal cancer [116]. Cho et al. demonstrated that rectal cancer while showing no difference in overall survival, did have a worse disease-free survival in the rectal cancer group. Importantly, they also demonstrated differences in the recurrence patterns between rectal and colon cancer, with rectal cancer tending to recur in the lungs as opposed to colon cancer’s predilection for the liver.

While widely believed to be of clinical benefit to colorectal cancer patients presenting with surgically amenable lung metastasis, the role of pulmonary metastasectomy continues to evolve. With no completed randomized clinical trials, surgeons must take into account the full body of literature when evaluating each patient’s candidacy for surgery.

### 4.3. Intraperitoneal Chemotherapy and Cytoreductive Surgery

With peritoneal spread found in 5 to 10% of patients on initial resection and 50% of patients with recurrent disease, treatment strategies aimed at improving the dismal survival of these patients are necessary [117,118,119]. Previously considered a diffuse metastatic disease because of its poor prognosis, a paradigm shift occurred when surgeons began to consider the peritoneal spread of colon cancer as a regional disease spread. With this change in philosophy came changes in treatment patterns suggesting that this disease could be locally resected with improvements in morbidity and survival.

**Figure 3:**
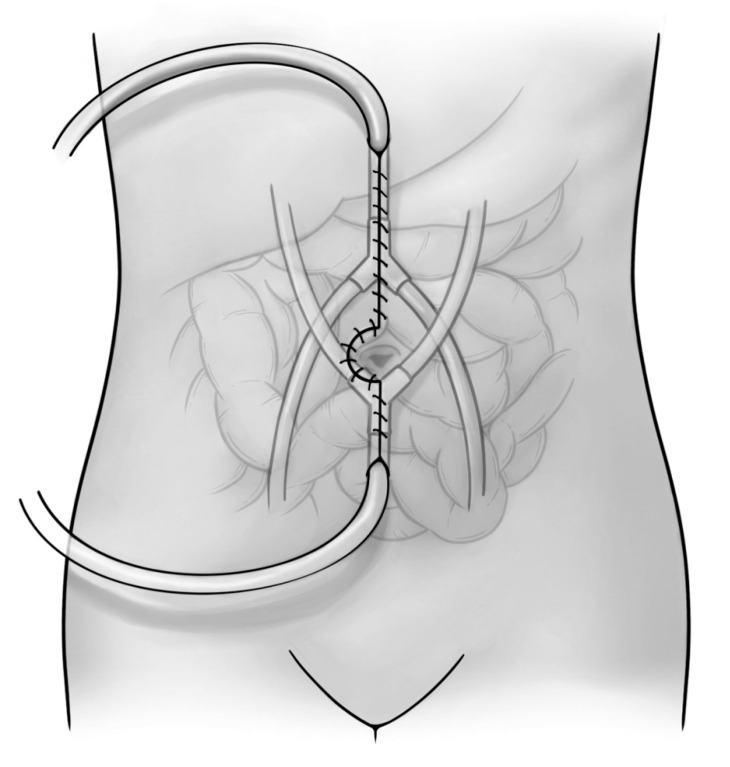
Heated intraoperative peritoneal chemotherapy administration.

With the introduction of cytoreduction and intraperitoneal chemotherapy to treat the peritoneal spread of colon cancer, intraperitoneal chemotherapy and cytoreductive surgery had undergone several vital improvements. In 1995, Dr. Sugarbaker introduced a stepwise approach to cytoreductive surgery capable of application in a variety of pathologies, giving rise to carcinomatosis. His approach recognized that the peritoneum was a poorly vascularized anatomical structure into which systemic chemotherapy had very limited penetration and efficacy. His approach outlined six separate peritonectomy procedures used in conjunction with intraperitoneal chemotherapy for the removal of localized metastatic disease to the peritoneum (Figure 3) [120].

While being utilized for years for the treatment of abdominal malignancies, the first randomized clinical trial for cytoreduction and hyperthermic intraperitoneal chemotherapy in patients with peritoneal carcinomatosis of colorectal cancer origin was completed in 2003 [121]. Verwaal et al. compared patients with peritoneal carcinomatosis from colorectal primary lesion over a 4-year span. These patients were randomized to cytoreduction and hyperthermic intraperitoneal chemotherapy with adjuvant postoperative chemotherapy or systemic chemotherapy alone without surgery. With the primary outcome of survival, the randomized clinical trial demonstrated survival improvements of 170% with the combination of surgical cytoreduction and intraperitoneal chemotherapy. A maximal benefit was observed in patients with localized disease in under five of the seven abdominal cavity compartments defined by Verwaal.

This stood for years as the primary cited evidence behind the treatment of colorectal peritoneal disease with surgical cytoreduction and HIPEC until Quénet et al. attempted to quantify each component of this combination therapy. Between the cytoreductive surgery component and the hyperthermic intraperitoneal chemotherapy component, Quénet et al. designed the multicenter PRODIGE7 trial to establish each individual component’s survival benefit [122]. Patients with Peritoneal Cancer Index (PCI) scores under 25 were randomized to cytoreductive surgery with or without oxaliplatin-based HIPEC. After a median follow-up of 63.8 months, their study demonstrated no significant survival or relapse-free survival differences between the cytoreductive surgery plus HIPEC group and the cytoreductive surgery group (41.7 vs. 41.2 months and 13.1 vs. 11.1 months). Surgical morbidity was statistically different between groups with a trend toward grade three or worse 30-day major complications in the combination HIPEC and cytoreductive surgery group (42% vs. 32%, *p =* 0.083). Overall, 30-day major complications were also statistically significant, with the HIPEC and cytoreductive surgery group having a higher rate of complications (26% vs. 15%, *p =* 0.035). From the totality of their 6-year study, they concluded that the addition of HIPEC to cytoreductive surgery conveyed no overall survival benefit and increased the risk of developing major postoperative late complications.

With non-invasive imaging strategies possessing disappointing detection rates in nodules smaller than 5 mm in diameter [123], and the high propensity of peritoneal carcinomatosis to derive from previously resected colorectal cancer, trials evaluating the need for “second-look” surgery to identify peritoneal recurrence were completed. As earlier, less invasive disease treated with cytoreductive surgery conveys a far better prognosis [124]. Goéré D et al. designed a randomized clinical trial attempting to quantify the benefit of early second-look surgery at 6-months after colorectal cancer resection [125]. He concluded early second-look surgery with high dose HIPEC oxaliplatin conveyed no advantage in disease-free or overall survival when compared to standard imaging surveillance. In addition, the patients who underwent second-look surgery with high dose HIPEC oxaliplatin had a 41% rate of grade 3–4 complications, highlighting that second-look surgery was not a simple risk-free procedure.

Previously thought to be a contraindication to HIPEC and cytoreductive surgery, what to do in the setting of concurrent peritoneal and liver metastasis has become a focus of recent study. Several studies have identified patients treated with HIPEC with cytoreduction and resection of their liver metastasis and compared their survival to their counterparts receiving modern systemic chemotherapy alone. While the chemotherapy regimen of these concurrent liver metastasis and peritoneal carcinomatosis patients differed between studies, the overall survival was demonstrated to be lower when compared to their isolated peritoneal carcinomatosis counterparts. This signified the poor prognostic outcome of this concurrent spread. When concurrent liver metastasis and peritoneal carcinomatosis patients who were treated with HIPEC, cytoreduction, and liver metastasectomy were compared to patients receiving traditional systemic chemotherapy, median overall survival was improved. This highlighted that this aggressive resection protocol may hold some potential benefit with concurrent liver and peritoneal spread [126]. This evidence, in combination with multiple systematic reviews, suggest that aggressive resection benefits this patient cohort and that liver metastasis should not be a contraindication for curative resection with HIPEC and cytoreduction [127,128]. However, without the presence of a well-designed randomized control trial, definitive recommendations cannot be made, highlighting the many questions left unanswered surrounding HIPEC and cytoreduction and its ripe potential for future study.

## 5. Conclusions

With the new and emerging treatment protocols for rectal cancer, it is paramount to have a full understanding of the current literature. It is important to understand in what patient population each tool in the colorectal surgeon’s armamentarium is ideally suited for. With this review, we highlighted transanal endoscopic microsurgery, transanal minimally invasive surgery, the “watch and wait” surveillance protocol, total neoadjuvant therapy, short-course radiation therapy, transanal and robotic total mesorectal excision, pulmonary and hepatic metastasectomy, and cytoreductive surgery with intraperitoneal chemotherapy with the hopes of bringing current research in the field to surgeons’ attention. Understanding these various therapeutic interventions will pave the way for improved patient outcomes moving forward and hopefully stimulate future innovation in the field.
